# Sediment accumulation by coastal biogenic structures sustains intertidal flats facing sea level rise in the German Wadden sea

**DOI:** 10.1038/s41598-025-03326-8

**Published:** 2025-05-27

**Authors:** Tom K. Hoffmann, Kai Pfennings, Jan Hitzegrad, Maike Paul, Achim Wehrmann, Nils Goseberg, Torsten Schlurmann

**Affiliations:** 1 Leibniz Universität Hannover, Ludwig Franzius Institute for Hydraulic, Estuarine and Coastal Engineering, Hannover, Germany; 2 Marine Research Department, Senckenberg am Meer, Wilhelmshaven, Germany; 3 Leichtweiß-Institute for Hydraulic Engineering and Water Resources, Technische Universität Braunschweig, Braunschweig, Germany; 4 Coastal Research Center, Joint Research Facility of Leibniz University Hannover and Technische Universität Braunschweig, Hannover, Germany

**Keywords:** Biogenic structures, Biogeomorphology, Drone mapping, Ecosystem engineer, *Magallana gigas*, *Mytilus edulis*, Environmental sciences, Hydrology, Ocean sciences, Geomorphology, Sedimentology

## Abstract

Biogenic structures such as oyster reefs and mussel beds can enhance sedimentation and promote the expansion of intertidal flats in the German Wadden Sea. This study presents large-scale measurements of sedimentation at biogenic structures that depend on biological activities, the hydrodynamic environment and surface roughness. As the surface roughness of intertidal flats has changed with the bioinvasion of the Pacific oyster and the resulting transformation of mussel beds into oyster reefs, we hypothesised that sedimentation rates vary between these structures. To quantify the extent of sedimentation over time, we surveyed bivalve-covered intertidal flats in 2020 and 2022 and generated digital models of the structures and surrounding terrain. The sediment accumulation rates for the mussel bed and oyster reefs reached 3.9 cm³/(cm²*y), with higher rates observed within the mussel bed than within the oyster reefs. Generally, biogenic structures at lower elevations experienced higher sediment accumulation rates. All accumulation rates exceeded previously reported rates for intertidal flats and the current rate of sea level rise in the Wadden Sea. Our findings suggest that oyster reefs and mussel beds have comparable effects on sediment accumulation, supporting the persistence of intertidal flats and thus helping to stabilise the Wadden Sea.

**Supplementary Information:**

The online version contains supplementary material available at 10.1038/s41598-025-03326-8.

## Introduction

The bioinvasion of the Pacific oyster (*Magallana gigas*^1^), has permanently changed the appearance of local intertidal flats in the German Wadden Sea over the past two decades, as this non-native species has transformed beds of the native blue mussel (*Mytilus edulis*^2^), into three-dimensional and rough reef structures^3–5^. Pacific oysters modify their physical environment in ways that shape ecological and geomorphological development, affecting both their survival and that of other species, consistent with the concept of niche construction^6^. This shift has caused irreversible ecological changes within the benthic habitat^7,8^ and may have altered local hydrodynamics, potentially impacting the surrounding sedimentation processes of fine-grained siliciclastics, organic material, biodeposits and the morphology of adjacent intertidal flats^9^. To date, blue mussel beds and oyster reefs, referred to below as biogenic structures, already cover up to 6% of tidal basin areas^8^, where the transformation has become relevant for coastal protection in the context of sea level rise (SLR), as the wave- and flow-damping effects of oysters and their contributions to sediment accumulation may exceed those of blue mussels^4^. We hypothesise that sediment accumulation in the vicinity of oyster reefs in the Wadden Sea has the potential to outpace local signals of anthropogenic SLR due to global warming, unlike the former blue mussel bed communities, which are much more sensitive to disturbances from severe storms and drift ice.

Various benthic species initiate biogeomorphological processes, shaping intertidal and coastal environments, where biological and geomorphological processes are inseparably linked^6^. With the presence of mussel beds, the common cockle (*Cerastoderma edule*) enhances sediment stability and microphytobenthos growth under high hydrodynamic stress. At the same time, lugworms (*Arenicola marina*) improve microphytobenthos by increasing sediment permeability and nutrient availability under deteriorated sediment conditions^10^. Flats with clams exhibit less stable seafloor conditions and greater erosion-accretion fluctuations, reducing the critical shear stress for erosion and increasing bed-level changes^11^. Bioturbators like the ragworm (*Hediste diversicolor*) and the peppery furrow shell (*Scrobicularia plana*) alter sedimentary and microbial dynamics depending on the season^12^. Given sufficient accommodation space, benthic species in intertidal systems can extend coastal wetlands, maintaining ecosystem resilience and mitigating large-scale losses of these vital ecosystems in coastal habitats^13^.

In particular, biogenic structures are recognised globally for enhancing coastal protection and facilitating sediment settlement and stabilisation^14–16^. However, orientation, height, elevation, flow velocity, and shear stresses influence sedimentation around and within these structures^17^; these morphological aspects of biogenic reefs have been vastly unaddressed by coastal science. When shear stress, consisting of components of tidal currents and wave oscillations, exceeds the critical shear stress, sediment can be eroded, transported, and deposited in the surrounding area^18,19^. On the one hand, elevated reef areas often experience relatively high flow velocities and turbulence, leading to erosion and sediment resuspension. On the other hand, they also reduce the flow velocity and enhance local sedimentation in the Wadden Sea^17^.

The German Wadden Sea, a unique and ecologically valuable coastal ecosystem, faces challenges from accelerated SLR^20^ and thus requires measures to preserve intertidal flats. While biogenic structures do not replace conventional or nature-promoted engineering measures^21^, they can complement coastal protection efforts as sustainable and cost-effective “green infrastructure”^4,22,23^. Biogenic structures support the protection of the hinterland by attenuating waves^22,24^ and promoting wave breaking, dissipating some initial sea state energy. However, strong wind fields over considerable distances across shallow tidal basins initiate a unique and counterintuitive process and re-energise the sea state, which leads to wave growth and increased shear stress, thereby increasing surge levels as they approach the coastline^25,26^. An excessively rapid SLR could intensify the impacts of local hydrodynamics, including coastal erosion and flood risks, as the local water depth increases^27–29^. In turn, this may put pressure on vulnerable coastal zones and pose significant risks to drowning intertidal flats and marine ecosystems^30,31^, with important ecological consequences^32^ such as losses of habitat, biodiversity, nutrients and natural resources. Numerical studies indicate that if sedimentation cannot compensate for SLR, the Wadden Sea may shift to a lagoon-like system with decreasing intertidal areas and altering ecological values^33–35^. Thus, sediment accumulation is essential for the persistence of tidal flats and counteracts the effects of SLR in shallow coastal environments such as the Wadden Sea^36^; this area serves as a role model for many other shallow coastal areas around the globe, hinting at the global processes behind biogenic reefs and their effects on morphological elevation adjustments to SLR.

Between 1993 and 2011, the observed local SLR ranged from 2.2 mm/y in the south (Norderney) to 6.6 mm/y in the north (Sylt) of the German Wadden Sea^37^. However, the Intergovernmental Panel for Climate Change (IPCC) projects accelerating SLR^38^, which, in turn, may surpass sediment accumulation rates and potentially drowning the Wadden Sea^20^. The interaction with the coastal geometry and bathymetry in back-barrier areas and ebb-tide deltas causes non-linear feedback effects on the SLR that influence tidal amplitudes and sedimentation processes^39,40^. Projections indicate an SLR of 0.8 m (median of RCP8.5) by 2100^41,42^. This means that the recent rate of SLR of 4.0 ± 1.53 mm/y^37^ will increase and potentially exceed the natural sediment accumulation^24,43^. Converting this projection, a simplified SLR rate will likely be between 10 and 20 mm/y by 2100 under RCP8.5^38^. Despite SLR, the Wadden Sea has shown resilience, with sediment accumulation rates ranging from 4 to 22 mm/y, averaging 9 mm/y between 1998 and 2016^43,44^, keeping pace with or even outpacing SLR^43,45,46^. These relatively high accumulation rates support the sustainability and vertical growth of the Wadden Sea and the maintenance of ecosystems, provided sufficient sediment is transported into the system and settles on intertidal flats^36,45^. However, as SLR tends to increase, sediment accumulation will become pivotal for intertidal flats to either sustain or drown at some point in the future. The Wadden Sea could benefit from the enhancement of sedimentation by ecosystems, such as biogenic structures.

Biogenic structures differ in coverage pattern, dimension, abundance, roughness level^47–49^ and influence on the surrounding hydro- and morphodynamics, resulting in varying sediment accumulation rates of ~ 0.7 cm per 2–3 months and 5 cm/y around and shoreward for oyster reefs^50,51^. The highest recorded rate of 29.0 cm/y was measured on the southeast Bangladesh coast^52^. Walles et al.^53^ identified a correlation between oyster reef length (the farthest extent of the reef outline perpendicular to the dominant wave direction) and geomorphological changes in the Oosterschelde estuary, indicating that reef dimensions influence areas of the same magnitude. For mussel beds, Brinke et al.^54^ reported sedimentation rates ranging from 5.0 to 10.0 cm during a summer in the same estuary. This observation aligns with Flemming and Delafontaine’s^55^ finding of sedimentation rates over 0.5 mm/day of accumulation during summer, while winter trends indicated minimal net deposition or erosion. The studies outlined above provide localised, point-specific acquired data on sediment accumulation rates around biogenic structures, but they omit insight into larger spatial scales. Investigating sedimentation dynamics across broader areas would provide a more general understanding of the magnitude of sedimentation provided by biogenic structures and whether they tend to enhance accumulation compared with plain, unsettled flats. Furthermore, direct comparisons are needed to examine whether oyster reefs may enhance sedimentation rates more effectively than mussel beds by quantifying sediment accumulation rates and vertical growth.

Our study seeks to fill a crucial research gap in understanding the geomorphological impacts of biogenic structures on sediment accumulation in coastal ecosystems, particularly in light of the ongoing transformation of native blue mussel beds into Pacific oyster reefs. As this ecological shift alters the roughness and morphology of intertidal flats, its effects on sedimentation are expected to change. Understanding this transformation is crucial for predicting future sedimentation patterns in the Wadden Sea and assessing the implications for coastal morphology.

We used the term accumulation to encompass all processes contributing to the increase in the sediment surface elevation. For simplicity, our focus is on the overall vertical growth of intertidal flats rather than delving into complex and interconnected processes. Even though location characteristics may also influence sedimentation processes, this study aims to capture general trends based on observations rather than conduct a detailed location-specific analysis. Further, we acknowledge that filter feeders, such as mussels and oysters, substantially contribute to accumulation through filtration and the biodeposition of pseudofeces. This contribution to accumulation depends on factors such as the filtration rate and abundance of the species, the relative water level and the inundation time. A key research question driving this investigation is whether non-native Pacific oysters are more effective than native blue mussels in promoting sediment accumulation and enhancing resilience against accelerated SLR in the German Wadden Sea. This research provides the first insights by analysing data from field observations from three representative locations and highlights the potential differences between the two species and their impacts on sediment dynamics. Gaining insights into the biogeomorphic role of these biogenic structures is vital for informing coastal management and conservation strategies in the Wadden Sea, with implications that extend to coastal environments worldwide.

## Study sites

**Fig. 1:**
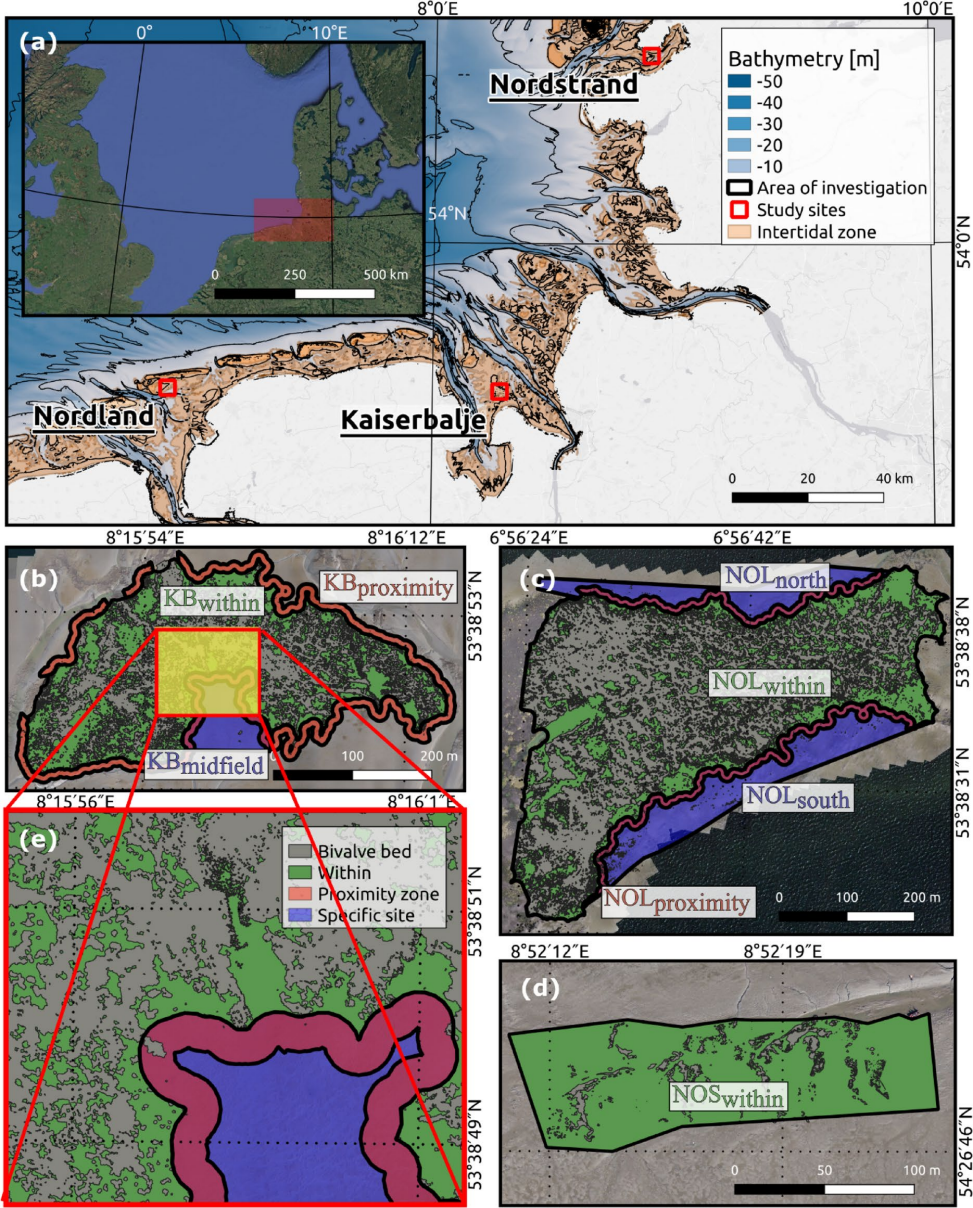
Map of the German North Sea showing the bathymetry^56^ and the locations of the study sites: Nordland (NOL), Nordstrand (NOS), and Kaiserbalje (KB) (**a**). The orthomosaics for KB (**b**), NOL (**c**), and NOS (**d**) show the analysed study sites and the areas of investigation (AOIs) within distinct boundaries (black solid lines). The green and red areas represent regions within and in a proximity zone (< 10 m) to the biogenic structures, respectively. The blue areas indicate specific sites, and the grey area marks the biogenic structures. Notably, the area of the KB is interrupted in the northwest, as tidal creeks run there; therefore, no reliable data are available. The enlarged subfigure exemplifies the distinction between the biogenic structures and the surrounding tidal flats (**e**). Map created with QGIS (version 3.16.16-Hannover, https://qgis.org*)* and Inkscape 1.1 (https://inkscape.org*).* Projection: ETRS89/UTM zone 32 N.

Our investigation focused on two oyster reefs, Kaiserbalje (KB) and Nordland (NOL), and one blue mussel bed, Nordstrand (NOS), in the German Wadden Sea (Fig. 1). The selection of study sites was based on the availability of well-documented biogenic structures monitored in previous studies^49,55^, ensuring accessibility and research continuity. We included one mussel bed that had not yet transformed to compare its sedimentation dynamics with those of established oyster reefs.

All three sites are characterised by fine sand to silt^43^ in the surrounding terrains. The oyster reefs are located in the Lower Saxon Wadden Sea: KB is 4.5 km north of the Butjadingen peninsula in Jade Bay and Outer Weser estuary, and NOL is 2.5 km south of the barrier island Juist. The first Pacific oysters were found at NOL in 1998 and KB in 2004^55,57^. Hence, the more mature reef at NOL had more time to develop and expand. Recently, average vertical median reef growth rates of 17.5 and 19.8 mm/y were determined for these reefs^49^. The NOS location is 800 m west of the mainland in the northern Wadden Sea. All three investigated sites are tidally influenced, mainly by the semidiurnal M2 tidal constituent. Maximum induced current velocities were generally between 0.2 and 0.4 m/s and rarely exceeded 0.5 m/s. Significant wave heights are mostly less than 0.5 m at all three locations, but the wave heights eventually increase during seasonal storm surge conditions with temporally elevated extreme sea levels due to wind-induced stresses^58^. The mean tidal ranges measured at the closest tide gauges are 3.55 m for KB (Hooksielplate), 2.39 m for NOL (Borkum Fischerbalje), and 3.50 m for NOS (Husum)^59^. Although the study areas exhibit similar hydrodynamic conditions, location-specific characteristics can influence biogeomorphological processes. Nevertheless, these specific factors are not considered within the scope of this research.

The study sites were divided into hereinafter defined areas of investigation (AOIs). The sediment budgets within the biogenic structures (KB_within_, NOL_within_, and NOS_within_) were analysed to obtain direct comparisons between the sites. A 10-meter proximity zone around the reef (KB_proximity_ and NOL_proximity_) was included to assess the influence of the structures on adjacent sedimentation and to capture potential spillover effects of sediment trapping beyond the biogenic structures. Furthermore, specific sites were selected on the basis of their possible role as settlement areas: KB_midfield_ served as a sediment trap because of its proximity to the surrounding oyster reef, whereas NOL_north_ and NOL_south_ were two locations positioned opposite each other and separated by an oyster reef. Since blue mussel coverage and the proximity zone varied greatly, only the zone within the NOS was considered.

## Results

### Sediment dynamics

#### Sedimentation rates

**Fig. 2:**
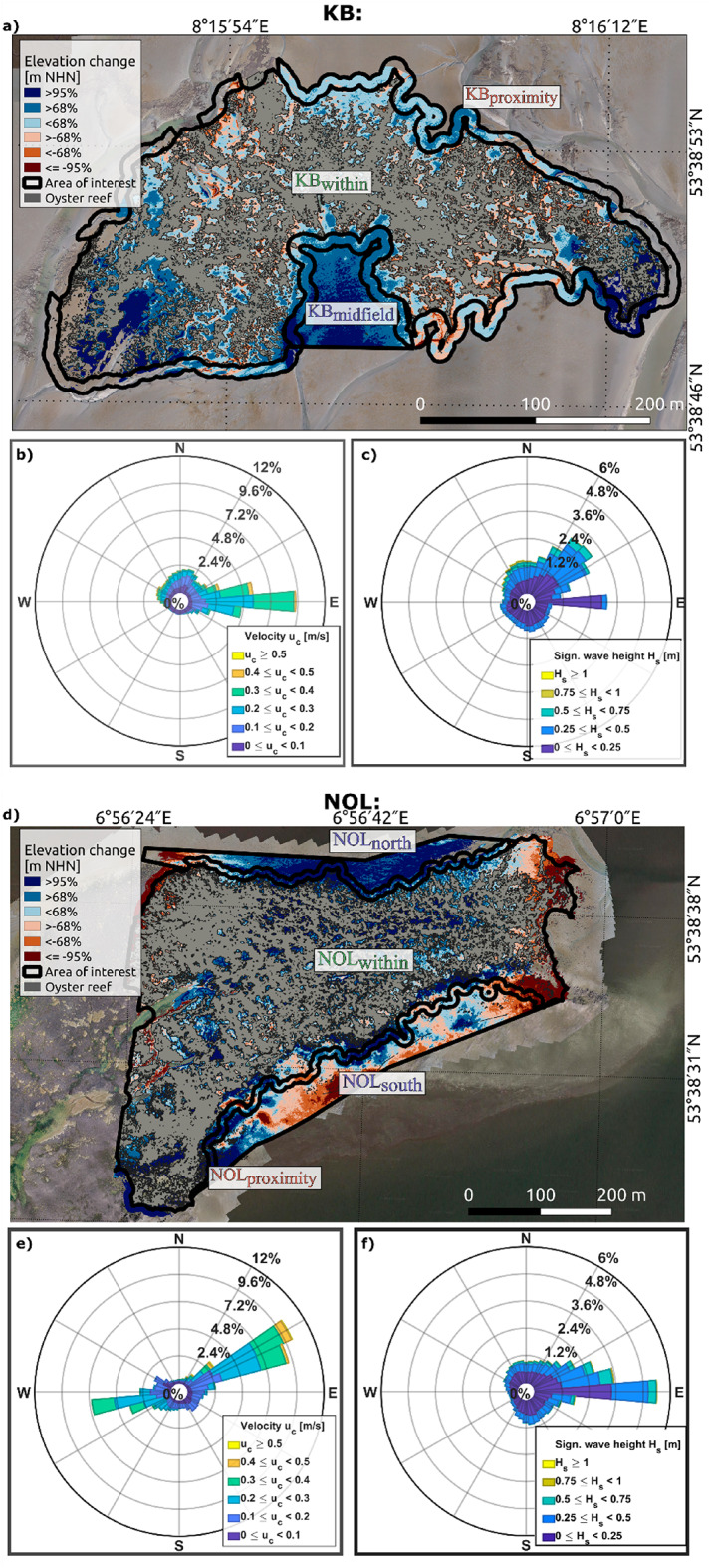

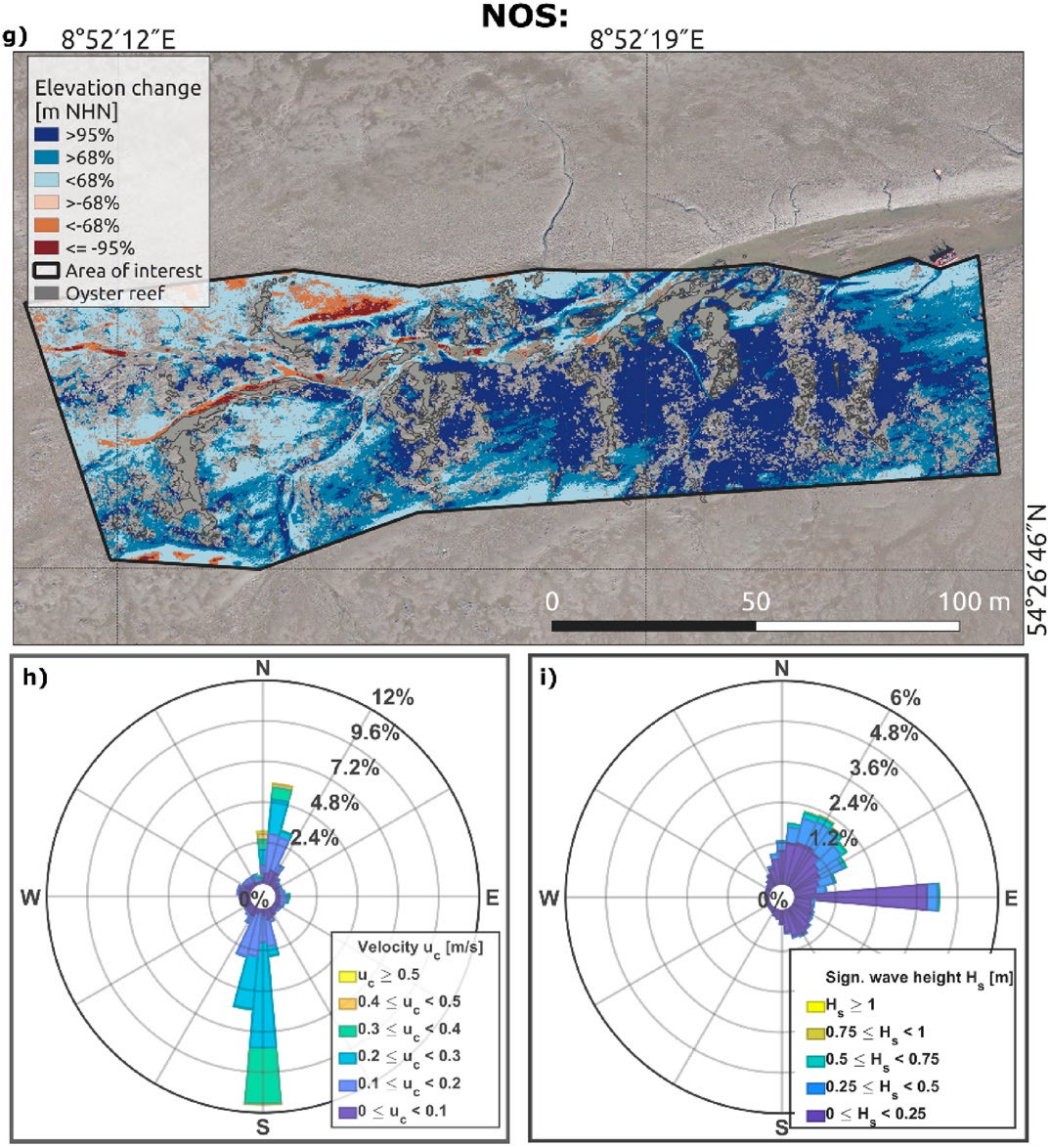
Total vertical changes in the sediment surfaces at the oyster reefs (**a** and **d**) and the blue mussel bed (**g**) between 2020 and 2022, divided into erosion (red-hued shades) and accumulation (blue-hued shades). The change detection results with a confidence level of 68% are presented in light blue and orange, and the changes with a confidence level of 95% are shown in dark blue and red. All topographical changes that do not consider uncertainties, and hence no confidence level, are presented with either a pale red or blue colour. The confidence levels correspond to a different minimum vertical change detection for each digital elevation model of difference (DoD). This study focuses on the results with a confidence level of 95%. Current and wave roses for each study site illustrate the direction, velocity magnitude, and frequency of currents (**b**, **e** and **h**) and the direction, significant wave height, and frequency of waves (**c**, **f** and **i**) during the period between 2005 and 2015^58^. DoDs created with QGIS (version 3.16.16-Hannover, https://qgis.org*).* Projection: ETRS89/UTM zone 32 N. Illustrations of rose diagrams generated with MATLAB 2023a (http://mathworks.com*).*

The study sites were monitored via unoccupied aerial vehicles (UAVs) in 2020 and 2022, providing high-resolution (< 5 cm/pixel) information on geomorphological changes. Volumetric changes were detected and quantified by balancing accumulation and erosion rates in the areas of investigation (AOIs) via digital elevation models of difference (DoDs). The focus was set on the significant changes (accumulation and erosion) with a 95% confidence level, corresponding to a minimum detection threshold between 8.7 and 9.0 cm (Table 1). Sediment accumulation with a 68% confidence level is additionally included in Fig. 2a, d and g (see also Supplementary Table 1). The influence of the confidence level on volumetric changes was discussed in Hoffmann et al.^60^.

**Table 1:** Vertical uncertainty of the GPS measurements *σ*_*GPS*_, validation of the alignment *σ*_*CP*_ and total vertical uncertainty of *σ*_*z*_ for 2020 and 2022. The vertical uncertainty of the corresponding DoD is shown in the last columns, with confidence levels of 68% (*σ*_*DoD*_) and 95% (*LoD*).

Survey	σ_GPS_ [cm]	σ_CP_ [cm]	σ_z_ [cm]	σ_DoD_ [cm]	LoD [cm]
KB					
2020 March	2.5	2.5	3.6	4.6	9.0
2022 March	2.2	1.9	2.9		
**NOL**					
2020 July	2.1	1.4	2.5	4.6	8.9
2022 April	2.5	2.8	3.8		
**NOS**					
2020 October	1.5	1.5	2.1	4.4	8.7
2022 March	2.8	2.6	3.9		

At KB, we observed distinct sedimentation and erosion patterns where volumetric changes across the area were unevenly distributed. Considering the 95% confidence level, significant accumulation primarily predominated in KB_midfield_ and the southern corners of KB_within_ (Fig. 2a), facilitated by the surrounding reef surfaces that probably weakened the hydrodynamics (Fig. 2b and c). Erosion occurred mainly along the reef edges and tidal creeks, enhanced by the relatively high flow velocities during the flood and ebb phases. In KB_within_, we recorded a large-scale accumulation of 124 m³ over an area of 2,810 m² (9% of the investigation area at the corresponding DoD without considering any confidence level) and minor erosion of approximately 2 m³ over 128 m² (< 1%). KB_midfield_ presented a consistent net sediment accumulation of 47 m³ over 2,995 m² (35%), with no substantial erosion. KB_proximity_ experienced an accumulation of 122 m³ over an area of 1,484 m² (11%).

At NOL (Fig. 2d), only the eastern part was analysed since this area has the most consistent UAV database. NOL_within_ and NOL_north_ primarily accumulated sediment, whereas erosion occurred in the eastern and southern parts. The eastern part is especially exposed to stronger currents and higher waves from the east (Fig. 2e and f), which may have facilitated erosion. The flats south of the reef experienced erosion and accumulation to the same extent, whereas the northern flats mainly grew. NOL_within_ experienced changes, with an accumulation of 552 m³ over 13,160 m² (27%) and erosion of 212 m³, occurring at the area’s eastern reef edge and the western boundary (4,505 m², 8%). NOL_north_ grew with an accumulation of 397 m³ in an area of 9,416 m² (57%), differing from NOL_south_, which balanced accumulation and erosion. NOL_proximity_ reached an accumulation of 170 m³ (4,072 m², 30%) and erosion of 7 m³ (340 m², 3%).

For NOS (Fig. 2g), a high volume of sediment accumulation was observed where NOS gained 139 m³ in an area of 3,415 m² (39%) and lost 2 m³ on 96 m² (1%). With respect to hydrodynamic forces, the alternating direction of tidal forces came from the south and north (Fig. 2h), whereas larger wave attacks were from the northeast, with smaller waves coming from the east (Fig. 2i). Note that although the hydrodynamic conditions influence local sedimentation, the corresponding data were not used for the analysis due to the low spatial resolution of 1,000 × 1,000 m and the low temporal resolution of annual intervals.

**Fig. 3:**
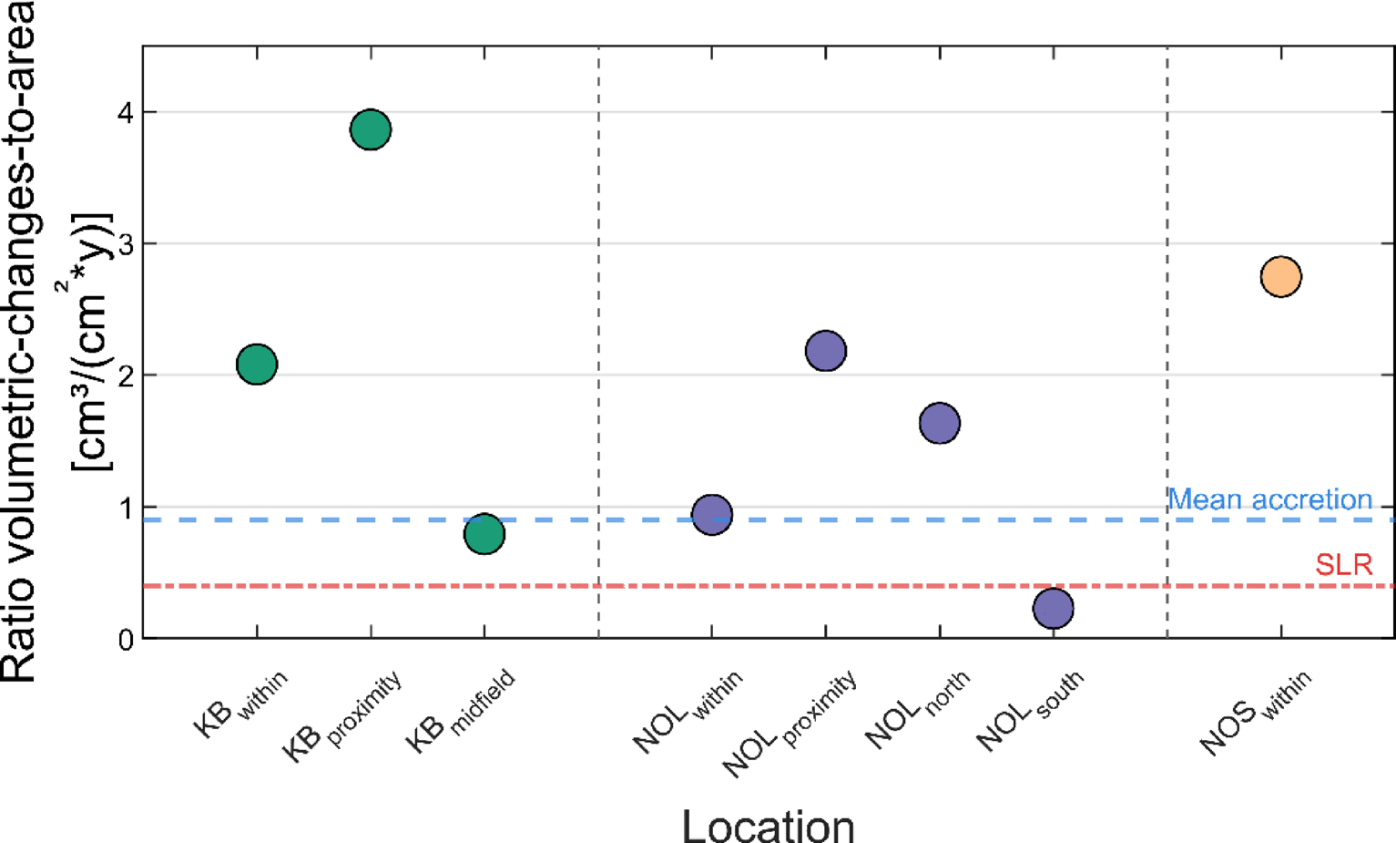
Annual sedimentation rates (cm³/(cm^2^*y)) over the study period. The significant changes include the vertical uncertainties, corresponding to a 95% confidence level. The red and blue lines illustrate the recent SLR (red) between 1993 and 2009^37^ and the general mean sediment accumulation (blue) of the intertidal flats between 1998 and 2016 in the German Wadden Sea^44^. Illustration generated with MATLAB 2023a (http://mathworks.com*).*

To account for variations in measurement periods across the study sites, the annual volumetric growth was standardised as a ratio of volumetric changes to the total area of significant change (cm³/(cm²*y)). It presents an accumulation rate with significant values of up to 3.9 cm³/(cm²*y) (Fig. 3). This metric reflects the annual sediment volume per unit area within regions of significant change and is calculated as the sum of the elevation differences between two DEMs that exceed the detection threshold (see Methodology). The accumulation rates highlight the sedimentation within and in the near field of biogenic structures, demonstrating that sediment accumulation surpasses erosion rates. Except for NOL_south_, all AOIs presented significant accumulation rates, exceeding the recent SLR rate and the general mean sediment accumulation^44^.

### Topographic changes

**Fig. 4:**
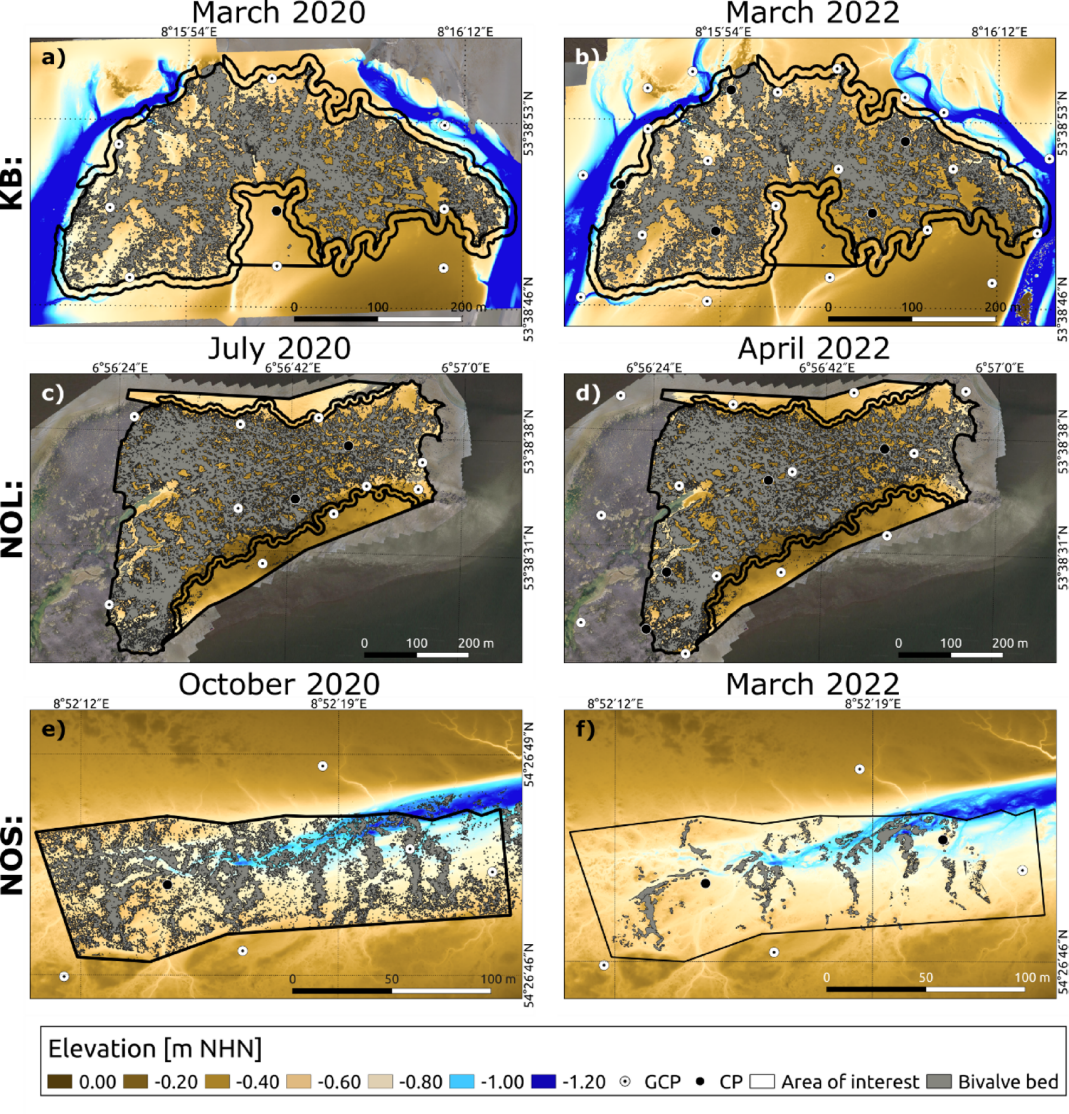
Digital elevation models (DEMs) of KB (**a** and **b**), NOL (**c** and **d**) and NOS (**e** and **f**) from 2020 (left side) and 2022 (right side). Created with QGIS (version 3.16.16-Hannover, https://qgis.org). Projection: ETRS89/UTM zone 32 N.

Volumetric changes with a confidence level may obscure potential growth patterns^60^. Thus, we also examined the sediment dimensions of the AOIs at the initial and final times to capture a complete picture of the sediment dynamics where no confidence level was considered (Table 2; Fig. 4).

**Table 2:** Area and volume (± error) of the sediment with the corresponding changes in percentages. The upper row indicates the values for 2020, and the lower row refers to 2022. The mean elevation is given with standard deviation (sd), and the maximum represents the 99.9% maximum elevation of the sediment surface elevation. All elevations are shown as standard elevation zero (NHN, vertical datum in Germany).

Study site	AOI	Area [m²] (2020) (2022)	Aerial changes [%]	Volume ± error [m³] (2020) (2022)		Volumetric changes [%]	Max. elevation [m NHN] (2020) (2022)	Mean elevation [m NHN] ± sd [m] (2020) (2022)	
Sediment									
**Within**	KB	32,755	3%	5,894	± 1,171	12%	-0.31	-0.52	± 0.09
		33,771		6,589	± 972		-0.27	-0.50	± 0.09
	NOL	60,192	-1%	16,024	± 1,532	15%	-0.30	-0.50	± 0.10
		59,778		18,450	± 2,256		-0.21	-0.47	± 0.11
	NOS	8,662	39%	2,780	± 136	67%	-0.38	-0.50	± 0.06
		12,002		4,650	± 464		-0.32	-0.42	± 0.06
**Proximity**	KB	13,377	11%	2,620	± 478	30%	0.00	-0.43	± 0.12
		14,910		3,398	± 429		0.02	-0.39	± 0.14
	NOL	13,356	6%	4,303	± 310	20%	-0.01	-0.38	± 0.17
		14,204		5,151	± 536		0.01	-0.34	± 0.16
**Specific sites**	KB	8,636	0%	1,756	± 309	39%	-0.38	-0.57	± 0.06
		8,637		2,437	± 249		-0.33	-0.50	± 0.07
	NOL	16,485	5%	2,080	± 325	68%	0.01	-0.27	± 0.12
		17,337		3,500	± 654		0.01	-0.27	± 0.12
	NOL	30,392	0%	12,966	± 774	1%	-0.30	-0.68	± 0.13
		30,535		13,110	± 1,153		-0.34	-0.62	± 0.13

At KB, KB_midfield_ exhibited the highest relative volumetric growth (~ 39%), likely because it was surrounded by the reef on three sides. KB_within_ and KB_proximity_ increased by approximately 12% and 30%, respectively. The largest relative volumetric growth was observed for NOL_north_ and NOS_within_, with values of approximately 68% and 67%, respectively. The areal sediment expansion of NOS_within_ caused strong growth in its sediment volume. In contrast, NOL_south_ did not increase.

The volumetric changes primarily encompass the vertical accumulation of sediments rather than changes in the surface area, except for the NOS_within_. Consequently, we also considered the sediment surface elevation in the analysis, representing the main factor in the volumetric changes.

Overall, NOL had the highest mean sediment surface elevation, significantly differing from KB and NOS for both 2020 (*p* < 0.001, F = 5,055,072, *n* = 36,059,679, Welch’s ANOVA) and 2022 (*p* < 0.001, F = 5,116,548, *n* = 42,147,854, Welch’s ANOVA). For KB and NOL, the mean elevation of the AOIs significantly differed in 2020 (KB: *p* < 0.001, F = 167,724, *n* = 21,907,182; NOL: *p* < 0.001, F = 17,451,602, *n* = 46,211,667, both Welch’s ANOVA) and in 2022 (*p* < 0.001, F = 2,459,717, *n* = 22,926,830; NOL: *p* < 0.001, F = 9,343,189, *n* = 48,741,423, both Welch’s ANOVA) (Fig. 4a-d). The Games–Howell post hoc test revealed statistically significant differences between all AOIs for all comparisons. The data further revealed a decreasing trend in elevation in the northerly direction at NOL (Fig. 4c and d). A comparison of the sediment elevation within the oyster reefs and the mussel beds revealed significant differences for both years: 2020 (t(3,992,251) = -3071.9, *p* < 0.001, *n* = 40,937,400, Welch’s t-test) and 2022 (t(4,304,207) = -2662, *p* < 0.001, *n* = 40,602,360, Welch’s t-test). While the areal coverage of the oyster reefs remained constant, the sediment area at NOS increased by 39%, probably due to a large loss (degradation) or local burial of the initial mussel bed (Fig. 4e and f).

**Fig. 5:**
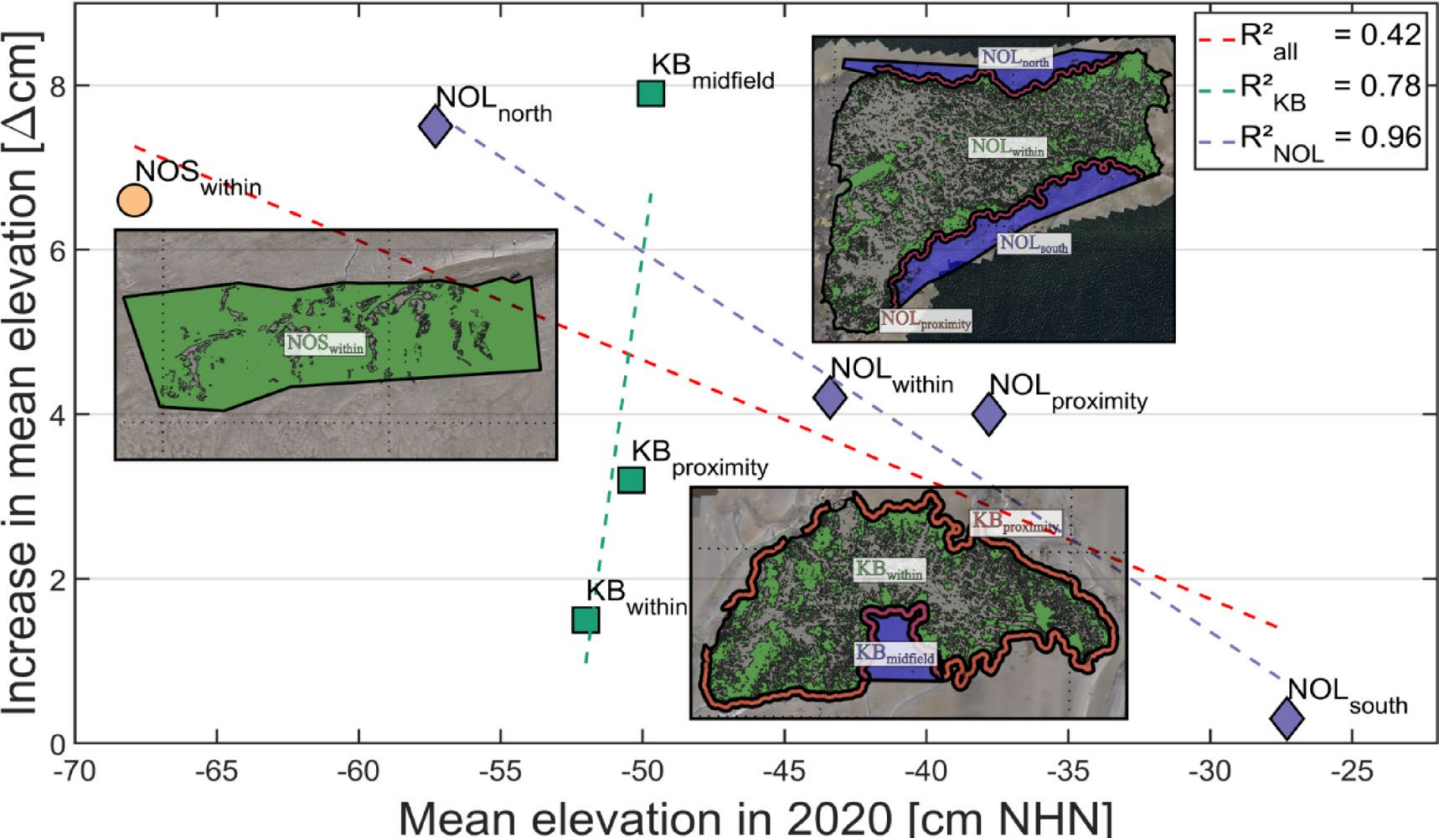
Correlation between the mean elevation (cm NHN) of all AOIs in 2020 and the increase in the mean elevation of the sediment surface (Δcm) between 2020 and 2022. The dashed lines indicate the linear best fit for NOL (purple), KB (green), and all study sites (red) with a corresponding coefficient of determination. While NOS is located further north at a lower mean elevation, NOL and KB in the southern German Wadden Sea are on higher sediment surfaces. Illustrations generated with MATLAB 2023a (http://mathworks.com*)* and Inkscape 1.1 (https://inkscape.org*).*

This study focuses on overall sediment accumulation as a comprehensive measure, offering a simplified perspective while encompassing all contributing mechanisms. To quantify the strength of the relationship between the mean elevation of all AOIs in 2020 and the increase in mean elevation (Δcm) from 2020 to 2022, the coefficient of determination *R²* is introduced. *R²* describes how well the variation in sediment accumulation can be linked to the variation in initial sediment elevation. The accumulation rates indicate a moderate correlation ($\:{R}_{all}^{2}$ = 0.42), where areas with lower elevations, such as NOS_within_ and NOL_north_, experienced greater sediment accumulation. The high mean sediment surface of NOL_south_ showed no noticeable growth (0.3 ± 4.6 cm), maintaining a mean elevation of approximately − 0.27 ± 0.12 m NHN. The highest $\:{R}^{2}$ correlation was determined for the AOIs of NOL, with a coefficient of $\:{R}_{NOL}^{2}$ = 0.96 (Fig. 5). In contrast, KB deviates from this trend, where the higher mean elevation increased the most (7.9 ± 4.6 cm at KB_midfield_), whereas the lower elevation increased the least (1.5 ± 4.6 cm at KB _within_). Since NOS consisted of only one AOI, no coefficient could be determined.

### Biogenic structures

The biogenic structures’ height and elevation (m NHN) relative to the mean water level impact sediment trapping and accretion within and near the beds. Within the time of observation between 2020 and 2022, an oyster coverage of almost 40,000 and 100,000 m² was detected by the machine learning algorithm *Random Forest* for KB_within_ and NOL_within_ (Fig. 1b and d), corresponding to 54% and 62% oyster coverage within the reefs, respectively. NOS showed a substantial decline in mussel coverage from 30 to 8% over the study period, where only the western part of the bed was analysed. Garland-shaped mussel patches (narrow but elongated structures similar to the structural class “garland” for oyster reefs described by Hitzegrad et al.^5^ for KB) below 5 m² were excluded because they were hardly detectable in this environment.

The oyster reef top of KB, defined as the maximum surface elevation, did not grow^60^, whereas the reef top elevation of NOL increased by 0.02 m. NOS had a lower top elevation than the oyster reefs (Table 3) and rose by 0.12 m from October 2020 to March 2022. Significant differences (*p* < 0.001, F = 1,121,808, *n* = 146,728,572, Welch’s ANOVA, Games-Howell test) were observed in the mean surface elevation between all three biogenic structures. For 2020 and 2022, the mean elevations of NOL were consistently higher than that of KB, and NOS had the lowest mean elevation.

**Table 3:** Descriptive statistics for the bivalve biogenic structures from the initial and final surveys of 2020 and 2022. N denotes the number of elevation data points. The mean elevation is given with standard deviation (sd), and the maximum represents the 99.9% maximum elevation of the biogenic structures. All elevations are given as standard elevation zero (NHN, vertical datum in Germany).

Study site	*n* [-] (2020) (2022)	Area [m²] (2020) (2022)	Aerial changes [%]	Max. elevation [m NHN] (2020) (2022)	Mean elevation [m NHN] ± sd [m] (2020) (2022)	
Biogenic structures						
KB	15,636,978	37,765	5%	-0.15	-0.40	± 0.10
	16,098,216	39,543		-0.15	-0.37	± 0.10
NOL	40,279,939	99,287	-2%	0.10	-0.34	± 0.11
	39,019,124	97,539		0.12	-0.29	± 0.12
NOS	1,213,363	4,013	-75%	-0.39	-0.67	± 0.13
	413,999	1,016		-0.27	-0.64	± 0.17

## Discussion

This study aimed to quantify sediment accumulation rates facilitated by biogenic structures in the German Wadden Sea and to compare their performance. Our large-scale quantification of vertical changes within and in the near field of biogenic structures, utilising high-resolution UAV monitoring and machine learning, provided robust and reliable results^60^ with a confidence level of 95% and highlighted areas of significant changes (Fig. 2).

Our results (Fig. 3) revealed accumulation rates of up to 3.9 cm³/(cm²*y). Although oyster reefs provide much more pronounced surface roughness than mussel beds do, NOS_within_ presented a higher accumulation rate of 2.7 cm³/(cm²*y) within its structures compared to the two oyster reefs, with 2.1 and 2.2 cm³/(cm²*y). Hence, we found no evidence that oyster reefs outperform mussel beds in terms of sediment accumulation within these structures. A potential reason for the unexpectedly higher sediment accumulation might be the lower surface elevation, creating a higher accommodation space and inundation time for sediment deposition^20,43^.

Overall, the sediment accumulation observed in this study could outperform the recent average rate of SLR of 0.4 ± 0.2 cm/y (1993–2009) in the North Sea^37^, except for NOL_south_ (Fig. 3) and support preserving intertidal flats when covered by biogenic structures. If sediment accumulation is lower than the local SLR or erosive conditions prevail, intertidal flats may become submerged, altering hydrodynamics and tidal properties^20^. The submersion of intertidal flats can substantially harm nature conservation by disrupting habitats vital for various marine and bird species^61^, necessitating adaptive measures to mitigate these processes. Even though accelerating SLR could outpace sediment accumulation rates and lead to the drowning of flats, it could also provide more sediment for accumulation on intertidal flats as long as sufficient sediment enters the system. A sufficient sediment supply to flats depends on the tidal range, inundation time and sediment pathways, whereas a decreasing inundation time and tidal range reduce the sediment load^20,43^.

All the AOIs presented accumulation rates, predominantly above the general mean accumulation of 0.9 cm³/(cm²*y) in the tidal basins of the German Wadden Sea, as deduced by Benninghoff and Winter^43^. The authors of^43^ reported rates between 0.4 and 2.2 cm³/(cm²*y), where individual tidal basins experienced accumulation rates of 0.8–1.0 cm³/(cm²*y) at KB, 0.5–0.8 cm³/(cm²*y) at NOL, and 1.0–1.3 cm³/(cm²*y) at NOS, without considering any existing biogenic structures. Both these and our results show that higher rates occur in the northern Wadden Sea than in the southern Wadden Sea, probably due to the greater accommodation space for sediment and longer inundation time^20,43^.

Notably, KB_midfield_, which is expected to be an effective sediment trap due to its surrounding oyster coverage and associated hydrodynamic damping effect, presented a lower accumulation rate of 0.8 cm³/(cm²*y). The introduced detection threshold, set at a 95% confidence level, may explain this result and highlight a potential limitation of the study, as only accumulation rates surpassing this threshold were considered. This may have led to an underestimation of overall sediment deposition, as smaller, evenly distributed increments may have gone undetected, decreasing this study’s accumulation rates.

Nonetheless, the resulting accumulation rates are consistent with those of previous studies by Thomsen and McGlathery^50^ and Southwell et al.^51^, who reported sedimentation rates of ~ 7 mm per 2–3 months and 5.0 cm³/(cm²*y) at reefs of the American oyster (*Crassostrea virginica)* in Virginia and Florida. Considering the far field, Chowdhury et al.^52^ quantified a maximum accumulation of 29.0 cm³/(cm²*y) over a distance of 35 m landward of the reef, which is distinctly greater than the results of this and other studies. This enormous discrepancy is probably due to the 0.8 m height of the artificially constructed oyster breakwater reef and its close proximity to the shoreline. Depending on species-specific behaviour and environmental conditions, such as temperature, certain benthic species can stabilise sediments^12^, while others enhance erosion^10^. For example, ragworms (*Hediste diversicolor*) promoted 1.2 cm of sediment accretion in winter through bioturbation, stabilising sediments, whereas peppery furrow shells (*Scrobicularia plana*) had minimal impact at colder temperatures due to reduced activity, leading to increased sediment resuspension^12^. In summer, *S. plana* intensified erosion, while ragworms continued to promote sediment stabilisation. In the presence of mussel beds, cockles (*Cerastoderma edule*) increased bed elevation by 4.6 cm over a year in sandy environments, whereas lugworms (*Arenicola marina*) facilitated erosion^10^. While these findings align with ours, they report slightly higher accumulation rates, likely due to the shorter study periods and seasonal effects not considered in our study. In contrast, our longer study period captures more robust results, reflecting longer-term consolidation processes. The higher accumulation rates in the other studies likely result from their shorter timeframes, emphasising the role of seasonal variations in sediment dynamics.

Our study demonstrates a general correlation between sediment accumulation and mean surface elevation where lower elevations promoted higher increases in the mean elevation. In contrast, high elevations experienced minimal vertical growth (Fig. 5). Splitting the AOIs by study site revealed a strong correlation for NOL, where the mean elevation increased less in the higher southern area, NOL_south_ than in the lower-lying northern area, NOL_north_. KB also demonstrated a high coefficient of determination (R) with a steep increase in mean elevation, rising from KB_within_ to KB_midfield_. However, the mean elevation of the AOIs at KB was so close to each other that no reliable correlation should be drawn.

Regarding the spatial extent of non-colonised sediment surfaces within biogenic structures, KB_within_ (+ 3.1%) and NOL_within_ (-0.7%) remained relatively stable over the two-year survey period. In contrast, NOS_within_ increased by 38.6% in sediment surface, indicating that either (i) larger areas surpassed the detection threshold of -0.9 m NHN, (ii) sediment started to cover the mussel bed, or (iii) mussel beds disappeared. Unlike oysters, which form rigid three-dimensional structures by cementing each other, mussel beds form a flexible meshwork on top of the sediment surface. They are, therefore, more vulnerable to ice drift and storm surges^62^. Severe storm events during the winter of 2021–22, notably in February 2022^63^, likely contributed to substantial losses in mussel bed coverage, as observed during field campaigns. However, severe winters that reduce the predator abundance of seabirds and crabs can favour recruitment, compensating for mussel bed losses with regular and successful recruitment events^64,65^.

Aside from hydrodynamic forces, the persistence of biogenic structures is also affected by sedimentation, which results in burial^17^. While oyster reefs, with their rigid structures, are more resistant to strong hydrodynamic stress and remain intact, mussel beds are more vulnerable to erosion and can be completely removed. Previous investigations at KB and NOL revealed that these oyster reefs are relatively constant in area (Table 3) and are experiencing vertical growth up to 19.8 mm/y^49,60^.

To better understand the biogeomorphological effects of biogenic structures on local sedimentation processes, future research should incorporate hydrodynamic data such as flow velocities and directions from field measurements that can be directly linked to sedimentation. Due to the limited spatio-temporal resolution of existing hydrodynamic data, these could not be integrated into our analysis yet. In addition, other location characteristics, such as sediment pathways or biotic factors, should also be considered as they may affect sedimentation processes.

The study sites represent only a small part of the intertidal zone of the central and northern Wadden Sea. This is the first study to measure and compare spatial sediment accumulation rates within and near two dominant types of biogenic structures, i.e., oyster reefs and mussel beds. However, direct comparisons between sediment accumulation rates in biogenic structures and uncolonised intertidal areas are lacking. Moreover, this investigation, which was conducted over two years, provides only a snapshot of the morphological changes at the study sites, and continuous monitoring is essential to track these changes and their implications. Understanding the further propagation of oyster reefs and the broader impact of biogenic structures on Wadden Sea morphology is crucial for developing effective persisting strategies and ensuring ecosystem resilience to future environmental changes. As ecosystem engineers, oysters provide biogenic structures that increase their ecological stability.

Furthermore, understanding the dynamics of oyster reef evolution over time can help leverage the adaptability of oyster reefs to environmental conditions. These insights could guide their role in enhancing sediment accumulation and eventually managing the evolution of intertidal flat landscapes on a larger scale, contributing to coastal protection measures that go beyond traditional line defences by integrating spatial protection concepts.

The persistence of intertidal flats is essential, as they provide valuable habitats and benthic communities. They act as natural wave breakers, reducing hydrodynamic forces on the shoreline while shaping tidal properties and water levels^24,43,46^. Our results suggest that biogenic structures may contribute to substantial sedimentation in the intertidal flats of the Wadden Sea despite accelerated SLR, which may exceed natural sedimentation rates in the future. This study revealed that since both species contribute to sedimentation to the same extent, transforming mussel-covered intertidal flats into oyster reefs could continue to support the persistence of the Wadden Sea ecosystem and form new hard substrate habitats for various organisms.

## Conclusion

This two-year investigation provides the large-scale measurement of sediment accumulation within and near oyster reefs and mussel beds, offering valuable insights into their impact on intertidal flat morphology. Although the study period represents a snapshot of long-term morphodynamic development, the accumulation rates of up to 3.9 cm³/(cm²*y) indicate that both biogenic structures contributed similarly to sediment accumulation from 2020 to 2022, exceeding previously determined accumulation rates between 0.4 and 2.2 cm³/(cm²*y) in the German Wadden Sea. This study captured general trends based on observations rather than conducting a detailed location-specific analysis. Biotic factors and location-specific factors, such as sediment surface elevation, accommodation space, and inundation time, also likely contribute to sedimentation, although their impact remains to be fully understood. Both ecosystems demonstrate that functional ecosystem services generate higher accumulation rates of 0.4 cm³/(cm²*y) than current and near-future SLR rates. We assume that sedimentation on biogenic structures will continue to be higher than the SLR in the near future, even if mussel beds turn into oyster reefs. This ongoing vertical growth will help preserve intertidal flats against SLR and support the stabilisation of the Wadden Sea.

## Methodology

The two oyster reefs and the blue mussel bed were surveyed by consumer-grade unoccupied aerial vehicles (UAVs, DJI Phantom 4 Pro), capturing and evaluating photo datasets around low water for each study site in 2020 and 2022. Access during low tide varied between surveys, which limited their overall spatial extent. Furthermore, only areas that were sufficiently covered by ground control points (GCPs) and check points (CPs) and represented on digital elevation models (DEMs) and orthomosaics in both years were analysed. The coordinates of the GCPs and CPs were measured via a Stonex-9000-dGPS with 0.8 cm horizontal accuracy and 1.5 cm vertical accuracy in real-time kinematic (RTK) network mode. The German standard datum, Normalhoehennull (NHN), defines the surface reference for elevation heights for mean sea level.

The UAV data allowed for high-resolution (ground sampling distance of ~ 1.2 cm/pix) photogrammetry and were aligned to six DEMs and orthomosaics via structure from motion (SfM), a reliable technique for generating digital models for subsequent spatial analysis and mapping of biogenic structures^5,60^, within Agisoft Metashape^®^ (1.7.3) for each survey. Afterwards, the machine learning algorithm *Random Forest*^66^ was used to classify the UAV data.

The overall uncertainty of the models *σ*_*z*_ was derived from the GPS measurements *σ*_*GPS*_ and the precision estimates of the CPs as validation points *σ*_*CP*_:

1 $$\:{\sigma\:}_{z}=\sqrt{{\sigma\:}_{GPS}^{2}+{\sigma\:}_{CP}^{2}}$$

All DEMs and orthomosaics were exported with a high resolution of 5 cm/pixel into QGIS (version 3.16 “Hannover”; qgis.org) to arrange the training dataset for classifying biogenic structures and sediment surfaces. This resolution was chosen as a compromise between computing power and accuracy. The training data were prepared and organised in QGIS and subsequently classified in R (v. 4.1.0) via RStudio (1.4.1717) and the randomForest package (v. 4.7–1.1). The training data included structural features of the DEMs (roughness, slope, and elevation), covering a scale of fine microtopography of the biogenic structures and spectral features from the RGB bands of the orthomosaics. The QGIS raster analysis tools determined the roughness as the degree of irregularity of the surface, whereby the largest difference between the cells of a central pixel and the surrounding cell was calculated. The slope is the angle of inclination to the horizontal. Based on the maps of detected biogenic structures, reef area *A* was determined from the total number of pixels $\:n$ of the classified raster layer of the DEM multiplied by the pixel area *A*_*pixel*_ (0.0025 m²):

2 $$\:A={n*A}_{\:}^{\:}$$

To determine the reef volume $\:{V}_{DEM}$ at the time of measurement, we summed the differences between the elevation of each pixel $\:{z}_{i}$ and the reference elevation $\:{z}_{ref}$ and then multiplied the sum by $\:{A}_{pixel}$:

3 $${V_{DEM}} = {A_{pixel}}\sum\limits_{i = 0}^{i = max} \vert{z_{ref}} - {z_i} \pm \:\sigma {\:_z}\vert$$

Areas below $\:{z}_{ref}$ = -0.7 m NHN, such as tidal creeks, were removed before processing as wet surfaces caused noise.

The volumetric changes were detected and quantified based on DEMs of difference (DoDs) by subtracting DEMs from each other pixel-by-pixel^67,68^:

4 $$\:DoD={DEM}_{2022}-D{EM}_{2020}$$

where positive values indicate an increase and negative values indicate a decrease in elevation. DoDs (Fig. 2) effectively visualise volumetric changes, considering uncertainties and highlighting significant erosion or accretion areas. To assign an uncertainty *σ*_*DoD*_ to the model, the error propagation was derived from two uncertainties *σ*_*z*_, corresponding to respective surveys following the method of Hoffmann et al.^60^:

5 $$\:{\sigma\:}_{DoD}=\sqrt{{\sigma\:}_{2020}^{2}+{\sigma\:}_{2022}^{2}}$$

for the DoD with a confidence level of 68%^69,70^. The level of detection (*LoD*) is a spatially consistent method introduced by Lane et al.^69^ to establish a threshold for significant geomorphological changes with a 95% confidence level. Here, $\:{\sigma\:}_{DoD}$ was multiplied by a statistical *t*-value of 1.96 under the t-distribution:

6 $$\:LoD=t*\sqrt{{\sigma\:}_{2020}^{2}+{\sigma\:}_{2022}^{2}}=t*{\sigma\:}_{DoD}$$

The *LoD* ensures the detection of statistically significant changes and is defined as the Root Mean Square Error (RMSE) of the total vertical uncertainties $\:{\sigma\:}_{z}$ for two DEMs. Although uncertainties might underestimate the results, this approach yielded growth trends with confidence. This approach is a simple and user-friendly technique for defining uncertainties (Table 1) that neglects the complexity of the composition of minor errors during recording and processing.

To determine the average annual volume increase per square centimetre (cm³/(cm²*y)), we calculated the total volumetric change by summing the height differences between the DEMs within the area of significant sediment change. This volume was then divided by the area of significant accumulation and erosion and divided by the time between the measurements to obtain an annualised accumulation rate.

### Statistics

The statistical analysis aimed to evaluate the variance in elevation of the sediment surfaces and biogenic structures across different AOIs and measurement time points using Welch’s ANOVA. Even though the elevation points of the DEMs, ranging from *n* = 3,454,604 to *n* = 24,076,974, exhibited a non-normal distribution, the large dataset allowed for robust statistical tests, making the distribution acceptable for analysis.

*Not a Number* (NaN) values and outliers were removed based on elevation thresholds, with values above 2 m NHN and below − 2 m NHN, to ensure the integrity and relevance of the dataset before analysis. The homogeneity of variances was evaluated via Levene’s test, which revealed heterogeneous elevation variances for all DEMs, making traditional ANOVA unsuitable. Thus, Welch’s ANOVA and Welch’s t-tests were applied to test the significance for groups of more than two or exactly two DEMs, respectively. Our analysis focused on the elevation of sediment surface or biogenic structures, the only response variable, making a multivariate test unnecessary. The biogenic structure type and study locations were treated as fixed factors, as these represent distinct, predefined categories. The sampling dates were considered random factors, as they were not systematically assigned but influenced by logistical constraints, such as tidal conditions and vessel availability. This approach allowed us to assess general trends in sediment accumulation while accounting for variability introduced by sampling dates. The Games Howell test was conducted as a post hoc test for pairwise comparisons if more than two groups were analysed. The significance level was set to α = 0.05 to determine statistical significance, and all tests were two-sided. All analyses were performed in R (version 4.3.3) via RStudio (2023.12.1 + 402, “Ocean Storm”), along with the packages raster (v. 3.6–26), car (v. 3.1-2), misty (0.6-2), psych (v. 2.4.3), rstatix (v. 0.7.2) and stats (v. 3.6.2).

## Electronic supplementary material

Below is the link to the electronic supplementary material.

Supplementary Material 1

## Data Availability

The datasets generated during and/or analysed during the current study are available in the PANGAEA repository, https://doi.pangaea.de/10.1594/PANGAEA.974854.
