# Supplementary material for: Sediment accumulation by coastal biogenic structures sustains intertidal flats facing sea level rise in the German Wadden sea
Source: Sci Rep. 2025 May 27;15:18518. doi: 10.1038/s41598-025-03326-8 (PMC12117113; doi:10.1038/s41598-025-03326-8)
Supplement: Supplementary file 1 — Supplementary Material 1 [file 41598_2025_3326_MOESM1_ESM.docx]

# Supplementary Material

**Supplementary Table 1.** Overview of accumulation and erosion for each area of investigation with confidence levels of 68% and 95%, where the RMSE is considered.

| **No consideration** | | | | | | | |
| --- | --- | --- | --- | --- | --- | --- | --- |
| **Area of investigation** | **Study site** | **Accumulated volume [m³]** | **Accumulated area [m²]** | **Eroded volume [m³]** | **Eroded area [m²]** | **Total growth [m³]** | **Yearly growth [cm³/(a*cm²)]** |
| Within | KB | 965 | 19,897 | -260 | 9,901 | 705 | 1.1 |
|  | NOL | 3,393 | 44,157 | -951 | 14,176 | 2,442 | 2.3 |
|  | NOS | 638 | 7,461 | -44 | 1,187 | 594 | 8.6 |
| Proximity zone | KB | 654 | 11,079 | -58 | 2,081 | 596 | 3.5 |
|  | NOL | 888 | 10,429 | -138 | 2,988 | 750 | 3.2 |
| Specific sites | KB: Midfield | 682 | 8,512 | -1 | 78 | 681 | 3.9 |
|  | NOL: North | 1,442 | 14,588 | -96 | 1,463 | 1,347 | 4.7 |
|  | NOL: South | 971 | 15,547 | -835 | 14,839 | 136 | 0.3 |
| **RMSE (68%)** | | | | | | | |
| **Area of investigation** | **Study site** | **Accumulated volume [m³]** | **Accumulated area [m²]** | **Eroded [m³]** | **Eroded area [m²]** | **Total growth [m³]** | **Yearly growth [cm³/(a*cm²)]** |
| Within | KB | 339 | 7,996 | -30 | 1,696 | 308 | 0.5 |
|  | NOL | 1,809 | 34,377 | -515 | 8,258 | 1,294 | 1.0 |
|  | NOS | 340 | 5,744 | -11 | 328 | 329 | 4.8 |
| Proximity zone | KB | 258 | 5,557 | -11 | 407 | 248 | 1.4 |
|  | NOL | 440 | 7,399 | -40 | 1,188 | 400 | 1.7 |
| Specific sites | KB: Midfield | 298 | 7,807 | 0 | 3 | 298 | 1.7 |
|  | NOL: North | 880 | 13,386 | -50 | 895 | 830 | 2.4 |
|  | NOL: South | 480 | 9,261 | -382 | 7,999 | 99 | 0.2 |
| **RMSE (95%)** | | | | | | | |
| **Area of investigation** | **Study site** | **Accumulated volume [m³]** | **Accumulated area [m²]** | **Eroded [m³]** | **Eroded area [m²]** | **Total growth [m³]** | **Yearly growth [cm³/(a*cm²)]** |
| Within | KB | 124 | 2,810 | -2 | 128 | 122 | 2.1 |
|  | NOL | 552 | 16,160 | -212 | 4,505 | 340 | 0.9 |
|  | NOS | 138 | 3,397 | -2 | 95 | 136 | 2.7 |
| Proximity zone | KB | 122 | 1,484 | -2 | 71 | 120 | 3.9 |
|  | NOL | 205 | 4,650 | -10 | 459 | 195 | 2.2 |
| Specific sites | KB: Midfield | 48 | 2,995 | 0 | 1 | 47 | 0.8 |
|  | NOL: North | 300 | 9,416 | -17 | 473 | 283 | 1.6 |
|  | NOL: South | 172 | 3,870 | -146 | 2,577 | 26 | 0.2 |
